# Bilayer graphene/HgCdTe based very long infrared photodetector with superior external quantum efficiency, responsivity, and detectivity †

**DOI:** 10.1039/c8ra07683a

**Published:** 2018-11-27

**Authors:** Shonak Bansal, Kuldeep Sharma, Prince Jain, Neha Sardana, Sanjeev Kumar, Neena Gupta, Arun K. Singh

**Affiliations:** Department of Electronics and Communication Engineering, Punjab Engineering College (Deemed to be University) Sector-12 Chandigarh-160012 India arun@pec.ac.in; Department of Metallurgical and Materials Engineering, Indian Institute of Technology Ropar India; Department of Applied Sciences, Punjab Engineering College (Deemed to be University) Sector-12 Chandigarh-160012 India

## Abstract

We present a high-performance bilayer graphene (BLG) and mercury cadmium telluride (Hg_1−*x*_Cd_*x*=0.1867_Te) heterojunction based very long wavelength infrared (VLWIR) conductive photodetector. The unique absorption properties of graphene enable a long carrier lifetime of charge carriers contributing to the carrier-multiplication due to impact ionization and, hence, large photocurrent and high quantum efficiency. The proposed p^+^-BLG/n-Hg_0.8133_Cd_0.1867_Te photodetector is characterized and analyzed in terms of different electrical and optical characteristic parameters using computer simulations. The obtained results are further validated by developing an analytical model based on drift-diffusion, tunneling and Chu's methods. The photodetector has demonstrated a superior performance including improved dark current density (∼1.75 × 10^−14^ µA cm^−2^), photocurrent density (∼8.33 µA cm^−2^), internal quantum efficiency (QE_int_ ∼ 99.49%), external quantum efficiency (QE_ext_ ∼ 89%), internal photocurrent responsivity (∼13.26 A W^−1^), external photocurrent responsivity (∼9.1 A W^−1^), noise equivalent power (∼8.3 × 10^−18^ W), total noise current (∼1.06 fA), signal to noise ratio (∼156.18 dB), 3 dB cut-off frequency (∼36.16 GHz), and response time of 9.4 ps at 77 K. Furthermore, the effects of different external biasing, light power intensity, and temperature are evaluated, suggesting a high QE_ext_ of 3337.70% with a bias of −0.5 V near room temperature.

## Abstract 2

We present a high-performance bilayer graphene (BLG) and mercury cadmium telluride (Hg_1−*x*_Cd_*x*=0.1867_Te) heterojunction based very long wavelength infrared (VLWIR) conductive photodetector.

## Graphical abstract

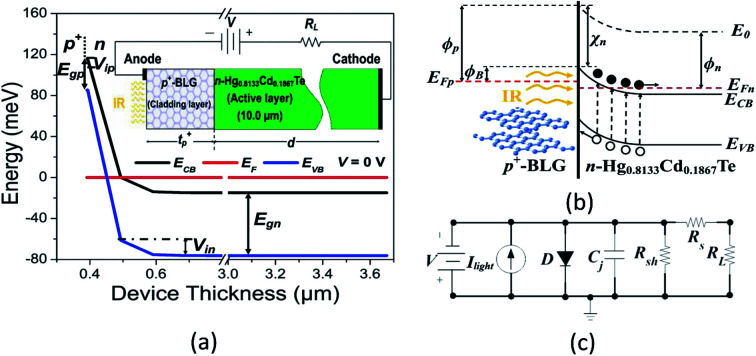

## Introduction

Infrared (IR) photodetectors have been successfully demonstrated from a wide variety of narrow bandgap semiconductors (Si and Ge)^1–3^ and alloys including III–V (InAs_1−*x*_Sb_*x*_, InAs, GaAs),^4–6^ IV–VI (Pb_1−*x*_Sn_*x*_Te),^4^ II–VI (Hg_1−*x*_Cd_*x*_Te, CdZnTe, CdSeTe).^4,7–10^ These materials absorb the incident photon energy corresponding to their bandgaps and result in the output photocurrent.^11^ InSb, a III–V compound semiconductor material was first used for IR detection. The IR detection region is categorized into short wavelength IR (SWIR: 1–3 µm), mid wavelength IR (MWIR: 3–5 µm), long wavelength IR (LWIR: 8–14 µm) and very long wavelength IR (VLWIR: 14–30 µm) ranges.^12^ The potential applications of IR photodetectors include biomedical and thermal imaging, gas sensing, night vision, spectroscopy, and free space communication *etc.*^4,13–15^ Rapid advances are being made in developing inexpensive narrow bandgap semiconductor photodetectors with improved sensitivity and longer wavelengths. As a result, low dimensional structures, including quantum-well, quantum-dot, and quantum-wire, based IR photodetectors are reported with improved performances, but with expensive fabrication processes.^16–19^ On the other hand, the low leakage current, tunable bandgap, better stability, low thermal generation rate and relatively high absorption coefficient make the ternary alloy mercury cadmium telluride (MCT: Hg_1−*x*_Cd_*x*_Te) a suitable material for high performance IR detectors.^12,14,20–22^ So far, numerous Hg_1−*x*_Cd_*x*_Te-based IR photodetectors with different configurations such as p–n,^23,24^ p–i–n,^14,25^ dual band IR detector,^15,26^ and avalanche photodiode^27^ have been reported at cryogenic temperatures. The high dark current limited by Auger recombination processes and low temperature operations are the major disadvantage of MCT based IR photodetector.^14,20,28,29^ Hence, there is a requirement to design and develop MCT based IR photodetectors demonstrating higher efficiencies near room temperature.

The excellent electrical and optical properties of graphene (Gr) enables utilisation of Gr as a transparent electrode integrated with conventional photodetectors for ultraviolet (UV) to IR regions.^30^ The Gr/semiconductor heterojunction based photodetectors are expected to demonstrate a low dark current, low power dissipation, small parasitics, higher breakdown voltage, and high response speed than that of conventional homostructures. The Gr has been successfully composited with different materials like ZnO,^31,32^ Si,^33^ CdS^34^ (for UV applications), CdSe,^35^ GaN^36^ (for visible applications), and Si,^1,37^ Ge,^3^ PbS,^38^ GaAs^39^ (for IR applications). However, the zero bandgap and small optical absorption (∼2.3%) of monolayer graphene (MLG) result in limited photocurrent responsivity (<1 A W^−1^) and photocurrent (*I*_light_) to dark current (*I*_dark_) ratios (*I*_light_/*I*_dark_).^6,30,33,40–42^ Accordingly, several efforts including utilisation of few layer graphene, Gr quantum dots, inducing small bandgap in Gr layers using either doping or under transverse electric fields are made to improve the charge injection and separation, suppressing dark current with improved efficiency.^11,13,43–46^ The intentional doping in monolayer and bilayer Gr shifts the Fermi-level either upward or downward, creating a bandgap opening.^47^ To the best of our knowledge, a maximum bandgap of 430 meV is found in p-doped bilayer graphene (BLG).^48^ Xu *et al.*^49^ demonstrated 25 times higher electrical conductance and optical transmittance of 80% from few layer (5–10) graphene transferred onto MCT substrate in the MWIR spectral region at 77 and 300 K. Despite such studies, no photodetector combining BLG with MCT is successfully studied yet. Hence, here we propose and simulate a BLG/MCT based photodetector providing excellent light absorption and electron transport. The device exhibits a low dark current and high temperature operation due to lower thermo-generation rate. In this work, we investigate the effect of different external bias, incident light power intensity and temperature on the performance of proposed heterojunction photodetector. It is evident from the results that high external quantum efficiency (QE_ext_) (>100%) is achieved due to long lifetime of photo-induced hot carriers in the VLWIR spectral region. The obtained results are further validated by developing an empirical model based on drift-diffusion, tunneling and Chu's methods in VLWIR, suggesting potential applications in next-generation high-performance, ultra-low-power, and cost-effective IR photodetectors for optoelectronics devices.

### Device structure and description

**Fig. 1:**
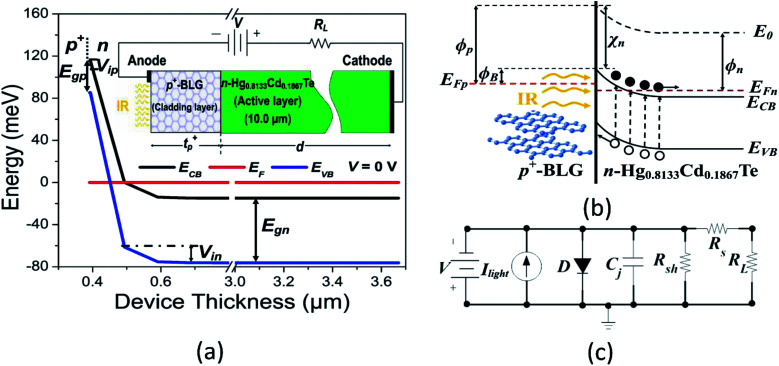
(a) The simulated energy bandgap diagram of p^+^-BLG/n-Hg_0.8133_Cd_0.1867_Te VLWIR photodetector at zero-bias (*V* = 0 V) under no illumination conditions. Here, *E*_CB_ and *E*_VB_ represent conduction and valence band energies, respectively; *E*_F_ is Fermi-level energy; *E*_gp_ and *E*_gn_ are the bandgap of p^*+*^- and n-regions, respectively; *V*_ip_ and *V*_in_ are the potential barriers in p^*+*^- and n-regions, respectively. The schematic of proposed photodetector is shown in the inset. The VLWIR radiations with an illumination cut-off wavelength of 20.6 µm are incident on the p^+^-BLG. Here, *t*_p^+^_ and *d* are the thickness of p^+^- and n-regions, respectively. *V* and *R*_L_ represent the applied bias and load resistance, respectively. (b) The schematic energy bandgap diagram of proposed photodetector under illumination and reverse bias condition. Here, *E*_0_ is the vacuum energy level; *E*_Fp_ and *E*_Fn_ are Fermi-level energies of p^*+*^- and n-regions, respectively; *ϕ*_B_ is the barrier height and *χ*_n_ is the electron affinity of the n-region. *ϕ*_p_ and *ϕ*_n_ denote the work function of p^+^- and n-regions, respectively. Under illumination the photo-induced carriers accumulate in the potential well, raising the Fermi-level and increasing the conductivity of the device. (c) The equivalent circuit of proposed BLG/MCT photodetector indicating photocurrent (*I*_light_), junction capacitance (*C*_j_), shunt resistance (*R*_sh_) and series resistance (*R*_s_) is utilised for evaluating dark current properties in Silvaco Mixed-Mode circuit simulations.

The inset of Fig. 1a shows the schematic of proposed BLG composite Hg_1−*x*_Cd_*x*=0.1867_Te based VLWIR photodetector. The highly doped p^+^-type BLG conformably cover 10.0 µm wide lightly doped n-type Hg_0.8133_Cd_0.1867_Te to form a heterojunction photodetector. The p^+^-doping of the Gr is typically being achieved through the chemical doping with HNO_3_,^50,51^ FeCl_3_,^52^ AuCl_3_,^53,54^ SOCl_2_,^55,56^ and NbCl_5_ (ref. 57) utilising chemical vapor deposition (CVD) technique. Practically, the Gr layers can be directly mechanically exfoliated or be transferred to suitable substrate utilizing Cu films in CVD growth techniques without any contamination issues.^58^ The electrical ohmic contacts of aluminium (Al) are made as anode and cathode to collect the photo-generated charge carriers. The nominal thickness of 2 nm for BLG is considered for our studies which is in consonance with previously reported values.^59–61^ The proposed p^+^-BLG/n-Hg_0.8133_Cd_0.1867_Te photodetector can be grown on lattice matched CdZnTe substrate. CdZnTe substrate is chosen because MCT/CdZnTe interface offers less interface trap charge as compared to Si, Ge, and CdTe substrates.^24^ In this paper, we have not considered the substrate effect, as it does not affect the device performance significantly. The Silvaco Atlas software is utilised to design and evaluate the electrical and optical performances of BLG/HgCdTe photodetector. The VLWIR radiations are incident from the p^+^-BLG cladding window over the narrow bandgap n-Hg_0.8133_Cd_0.1867_Te active/absorbing layer. The p^+^-BLG is used as a light absorber. The lightly doped n-Hg_0.8133_Cd_0.1867_Te generates total dark current and photocurrent. The bandgap and doping concentration of active region are selected to give better absorption of VLWIR radiations suppressing thermally generated carriers. In order to simulate the device, a bandgap of 250 meV for BLG is considered.^62,63^ When VLWIR light illuminates the p^+^-BLG/n-Hg_0.8133_Cd_0.1867_Te heterojunction, the hot photo-carriers transport from the active layer to the Gr layer due to built-in electric field (*E*_built-in_).^64^ As a result, the change in graphene conduction is observed according to the relation, Δ*σ* = Δ*nqµ*,^65,66^ where Δ*n* represents variation in carrier-concentration of graphene; *q* corresponds to the electronic charge; and *µ* is the carrier mobility. The schematic illustration of depletion region with a barrier height (*ϕ*_B_) between the BLG and HgCdTe is given in Fig. 1b. The presence of potential barrier within the heterojunction is due to the existence of gradients in electron affinity ∇*χ* and electrostatic potential ∇*V*. The electrons are dominated charge carriers than holes. The electrons travel from n-region into the lower energy states in p^+^-region, leaving the positively charged empty states in the n-region. Accordingly, the energy band bends upward in the vicinity of the p^+^–n heterojunction to form an *E*_built-in_. The doping profile of the material determines the barrier height, however, potential variation results in energy band drop at p^+^–n interface junction^23^ as shown in Fig. 1a and b. The existence of the small bandgap in Gr due to doping will change the work function of Gr resulting in shifting of Fermi-level towards the conduction band. This makes graphene different than the others 2D semiconductor materials.^67^ It is also observed from Fig. 1b that under illumination, the photo-induced carriers accumulate in the potential well, raising the Fermi-level and increasing the conductivity of the device. The energy band diagram of the device can be explained by the classical theory of Anderson.^68^ The absorbed photo carriers undergo hot carrier multiplication (CM) by impact ionization (also known as inverse Auger recombination process)^69–72^ in p^+^-BLG, subsequently cross the barrier through internal photoemission (IPE) process (in picosecond scale),^33^ and finally be collected by n-Hg_0.8133_Cd_0.1867_Te contributing the total photocurrent. The carrier multiplication factor can further be tuned with chemical doping.^73,74^ The fast recombination rate (in picosecond) of the photogenerated carriers results in a maximum multiplication factor of 4.3 by the impact ionization process.^75^ Thus carrier multiplication, *i.e.* internal gain mechanism in BLG will enhance the photocurrent *I*_light_ of the device due to the increase in carriers.^76^ The increase in photocurrent will result in higher internal quantum efficiency (QE_int_),^33^ facilitates to break through the upper-limit of traditional MCT based IR photodetectors. This indicate that our BLG composite photodetectors can still work effectively under a low illumination conditions. In our device, we have achieved a carrier multiplication factor of 1.25 for p^+^-doped graphene at 250 K, which is in good agreement to the earlier reported values.^73,74^ In addition, an equivalent Mixed-Mode circuit is utilised for estimating device performances in dark condition as shown in Fig. 1c. It consists of an equivalent diode in parallel with shunt resistance *R*_sh_, junction capacitance *C*_j_, and load resistance *R*_L_ with series resistance *R*_s_ at an applied bias *V* for generating carriers contributing to the device current. The effect of illumination is implemented by a constant current source *I*_light_ in Silvaco Mixed-Mode simulator.

In self-powered mode, the depletion layer width *w* = [2*ε*_0_*ε*_r1_*ε*_r2_(*V*_bi_ − *V*)(*N*_A_^2^ + *N*_D_^2^)/(*q*(*ε*_r1_*N*_A_ + *ε*_r2_*N*_D_)*N*_A_*N*_D_)]^1/2^ of the heterojunction is found to be 64.28 nm for a built-in potential (*V*_bi_ = *V*_ip_ + *V*_in_) of 21 mV. Here, *V*_ip_ and *V*_in_ are the barriers corresponding to energy band bending at p^+^- and n-regions, respectively. *ε*_0_ is the absolute permittivity and *ε*_r1_ & *ε*_r2_ represent the relative permittivity in p^+^- and n-regions, respectively. *N*_A_ (2 × 10^22^ cm^−3^) and *N*_D_ (1 × 10^16^ cm^−3^) corresponds to the acceptor and donor doping concentrations in p^+^- and n-regions, respectively. The thickness of bilayer graphene is considered to be 2.0 nm, which is considerably smaller than that of MCT (10.0 µm). Accordingly, the depletion region extends inside the MCT (n-side) resulting in gradual shrinkage in band diagram towards BLG/MCT interface as observed in Fig. 1a employing computer simulations. The junction capacitance *C*_j_ = *ε*_0_*ε*_r2_*A*/*w* in self-powered mode is found to be 0.98 fF. The device exhibits the transit time *τ*_*t*_r__ = *w*/*v*_satn_ of 6.4 ps, where *v*_satn_ is the carrier saturation velocity (10^6^ cm s^−1^) in n-region. The dependence of *ε*_r2_ on *x* composition for n-region is approximated by:^12^

1 *ε*_r2_(*x*) = 20.5 − 15.6*x* + 5.7*x*^2^

## Results and discussions

### Electrical characterization

The p^+^-BLG/n-Hg_0.8133_Cd_0.1867_Te photodetector is electrically characterized by solving continuity, carrier transport and Poisson equations with optimized boundary conditions as approximated by Boltzmann's transport model.^15^ Here, BLG is considered as 3D in nature analogous to earlier reported studies demonstrating the integration of multilayer graphene with other conventional semiconductors which couples 2D transport equations to 3D equations.^67^ A 2D semiconductor material has one physical dimension of the order of Fermi wavelength *λ*_F_. In our case, 
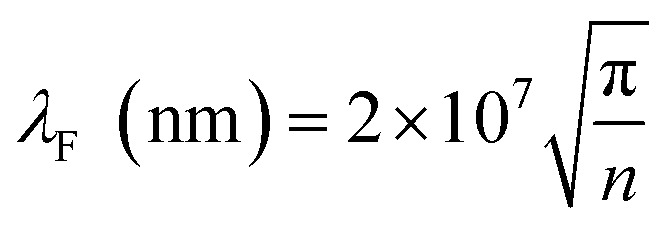
 of graphene is estimated to be around 0.56 nm for the sheet carrier density (*n*) of 4 × 10^15^ cm^−2^. The obtained value of *λ*_F_ is smaller than the considered thickness of doped BLG (2 nm), hence, it is necessary to consider 3D equations for estimating device characteristics. Further, to reproduce the carrier transport in graphene a drift-diffusion approach is implemented to degenerate semiconductor and parabolic shape of the conduction band.^77^ The Newton–Richardson iteration method and concentration dependent Analytic model are used to estimate the carrier mobility in the photodetector.^15,24^ In order to characterize the carrier lifetime and the dark current in the proposed photodetector, Shockley–Read–Hall (SRH), Auger, optical (band-to-band), trap assisted tunneling (TAT), and band-to-band (BTB) standard tunneling mechanism models are considered. The doping and carrier densities are evaluated using Fermi–Dirac statistics.^15,24,78^

The performance of the photodetector at 77 K is evaluated analytically for the operation at the wavelength of 20.6 µm. The photons having energy greater than the bandgap create electron–hole pairs in the lightly doped n-region. The mole fraction *x* of cadmium in the Hg_1−*x*_Cd_*x*_Te tunes the energy bandgap in VLWIR with a cut-off wavelength of 20.6 µm. For n-Hg_1−*x*_Cd_*x*_Te, the energy bandgap (*E*_gn_), electron affinity (*χ*_n_), and intrinsic carrier concentration (*n*_in_) as a function of *x* composition and lattice temperature *T* are approximated by eqn (2), (3) and (4), respectively.^12,24,79^2
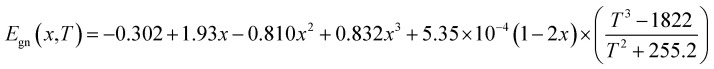

3 *χ*_n_(*x*,*T*) = 4.23 − 0.813 × [*E*_g_(*x*,*T*) − 0.083]

4
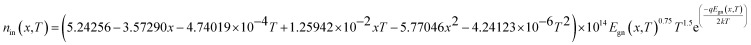
where, *k* is the Boltzmann constant.

**Fig. 2:**
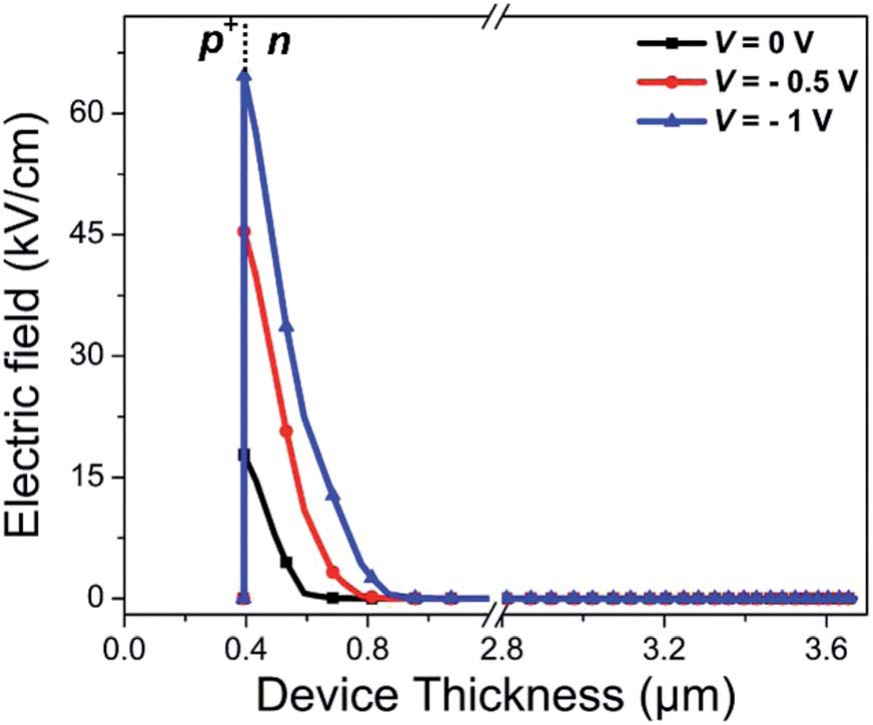
The electric field profile across the device at different external biasing conditions demonstrate high electric field at the p^+^–n heterojunction of BLG and MCT.

The electron effective mass 
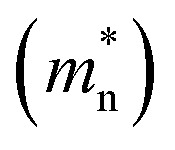
 of n-Hg_1−*x*_Cd_*x*_Te material is estimated by using Weiler's expression^80^ (eqn S1 and S2†). The various computation parameters based on previous studies including effective density of states for electrons (*N*_CB_) and holes (*N*_VB_) in conduction and valence bands, respectively, intrinsic carrier concentration (*n*_i_), and carrier (electron and hole) lifetimes are listed in Table 1.^12,24,67,81^Fig. 2 shows the triangular shape built-in electric field profile across p^+^–n heterojunction VLWIR photodetector for different biasing conditions. The maximum value of electric field (*E*_max_) is found to be 17.8, 45.4, and 64.6 kV cm^−1^ at the heterojunction of the device with an external bias of 0, −0.5, and −1 V, respectively. The electric field *E*(*x*) across the device can be written as:5
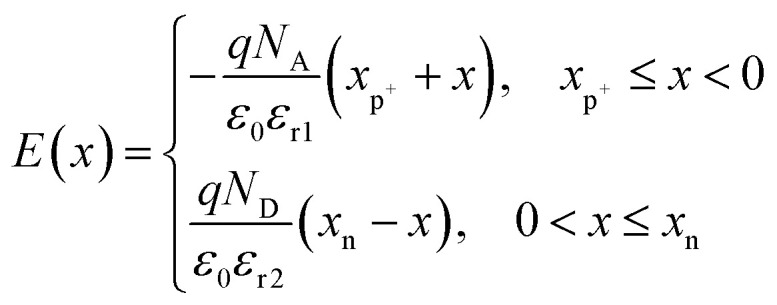
where *x*_p^+^_ and *x*_n_ are the depletion width in p^*+*^- and n-regions, respectively.

**Table 1:** Optimised simulation parameters used for device analysis

Parameters	p^+^-BLG	n-Hg_1–*x*_Cd_*x*_Te
*x* Composition	—	0.1867 (cal.)^24^
Bandgap (meV)	(250)^67,81^	60.2 (cal.)^24^
Affinity (*χ*) (eV)	(4.2)^67,81^	4.25 (cal.)^24,79^
Relative permittivity (*ε*_r_)	(3.3)^67,81^	17.8 (cal.)^12,24^
*N*_CB_ (cm^−3^)	(5.2 × 10^16^)^67,81^	1.0285 × 10^15^ (cal.)^79^
*N*_VB_ (cm^−3^)	(5.2 × 10^16^)^67,81^	1.3310 × 10^18^ (cal.)^79^
*n*_i_ (cm^−3^)	3.4312 × 10^8^ (cal.)^79^	3.8213 × 10^14^ (cal.)^24^
*N*_A_ (cm^−3^)	2 × 10^22^ (assumed)	—
*N*_D_ (cm^−3^)	—	1 × 10^16^ (assumed)

BLG is atomically thin with a thickness of the order of nm. This may create the depletion region width *x*_p^+^_ either almost fully depleted or very thin (≈3.21 × 10^−16^ m in our case), hence, can be safely neglected. The electron–hole pairs from MCT can be effectively separated by high built-in *E*_built-in_ to generate the photocurrent. The dark current density (*J*_dark_) is a combination of diffusion current density (*J*_DIFF_) in the neutral p^+^- and n-regions, drift current density (*J*_GR_) due to generation–recombination of charge carriers in the depletion region and the tunneling current density (*J*_TUN_). *J*_TUN_ consists of the contribution of both the trap-assisted tunneling (TAT) and band-to-band (BTB) tunneling. Accordingly, the total dark current density as a function of voltage and ambient temperature is given by:

6 *J*_dark_(*V*,*T*) = *J*_DIFF_ + *J*_GR_ + *J*_TAT_ + *J*_BTB_

The total diffusion current density is evaluated as:^24^

7 *J*_DIFF_ = [(*J*_p_)_n_ + (*J*_n_)_p_](e^(*qV*/*kT*)^ − 1)

where *V* represents the applied bias voltage, the diffusion component of current density for holes (*J*_p_)_n_ and electrons (*J*_n_)_p_ in n- and p^+^-regions, respectively, are given as:8
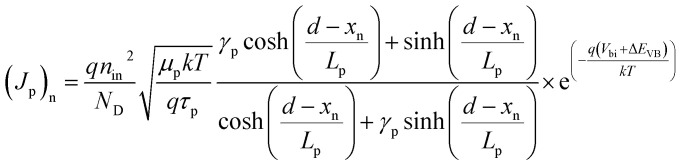
9
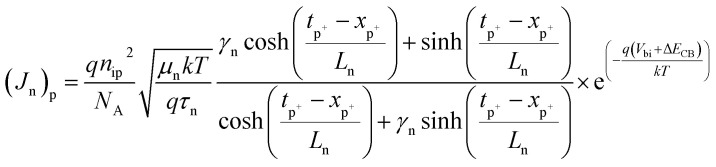
where *n*_in_ and *n*_ip_ are the intrinsic carrier concentrations in n- and p^+^-regions, respectively. *µ*_p_ and *µ*_n_ are the hole and electron mobilities, respectively. *τ*_p_ and *τ*_n_ represent the hole and electron lifetimes, respectively. *γ*_p_ = *S*_p_*L*_p_/*D*_p_ and *γ*_n_ = *S*_n_*L*_n_/*D*_n_ are the ratios of surface to bulk recombination velocities in p^+^- and n-regions, respectively. Here, *S*_p_ = 10^3^ cm s^−1^ and *S*_n_ = 10^5^ cm s^−1^ are the surface recombination velocities of holes and electrons, respectively at the heterojunction.^24^
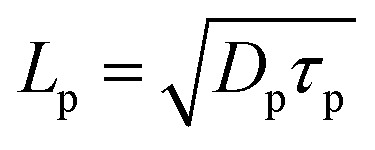
 and 
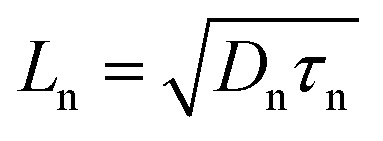
 are the diffusion lengths of holes and electrons. *D*_p_ = *µ*_p_*kT*/*q* and *D*_n_ = *µ*_n_*kT*/*q* represent the diffusion coefficients of holes and electrons, respectively. The valence and conduction band-edge discontinuities, after the formation of the p^+^–n heterojunction are given by Δ*E*_VB_ (=Δ*E*_g_ − Δ*E*_CB_) and Δ*E*_CB_ (=*χ*_n_ − *χ*_p_). *χ*_n_ and *χ*_p_ are the electron affinities of the wide and narrow bandgap materials, where *χ*_n_ > *χ*_p_. Δ*E*_g_ (=*E*_gp_ − *E*_gn_) is total bandgap discontinuity and *E*_gp_ and *E*_gn_ represent the energy bandgaps in the p^+^- and n-regions.

The transfer of charge carriers across the p^+^–n heterojunction is strongly affected by the trap levels at the heterojunction particularly in depletion region. The electron and the hole components of current density due to the generation–recombination in the depletion region is given as:^82^10
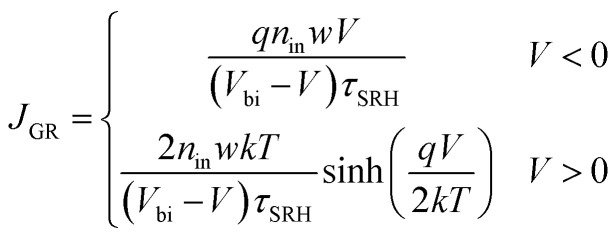
where *τ*_SRH_ = 1/*σN*_f_*v*_th_ is SRH generation–recombination lifetime of carriers. *σ* = 6.9591 × 10^−16^ cm^2^ represents the capture cross section of minority carriers. *N*_f_ is the SRH trap density. 
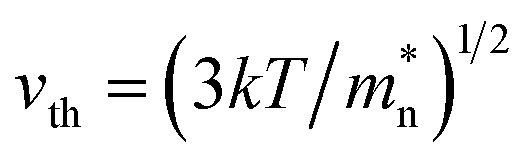
 is the thermal velocity of minority carriers.

The current related to TAT corresponds to the tunneling of electrons to the conduction band *via* a trap level within the bandgap. These trap states are the intermediate energy levels generated due to the existence of impurities in the material. The TAT component of current density is evaluated as:^82^11
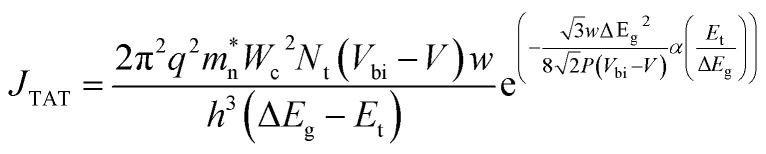
where12
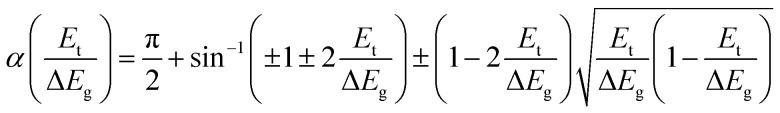
Here, *N*_t_ is the trap density different from SRH trap density, and *h* is the Planck's constant. *W*_c_ and *P* represent matrix elements associated with the potential and interband matrix elements, respectively. The position of the trap levels in the bandgap is indicated by *E*_t_.

The high reverse bias causes bending in energy band to tunnel the electrons from valence band to the conduction band as shown in Fig. 1. This BTB current density is given by:^82^13
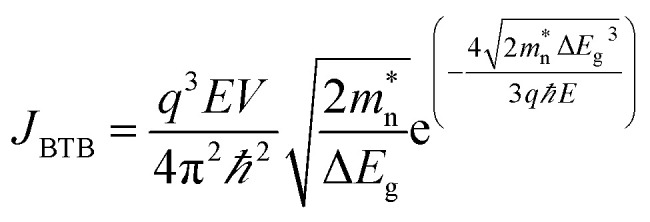
where *ℏ* = *h*/2π is the reduced Planck's constant, *E* represents the electric field across the depletion region.

**Fig. 3:**
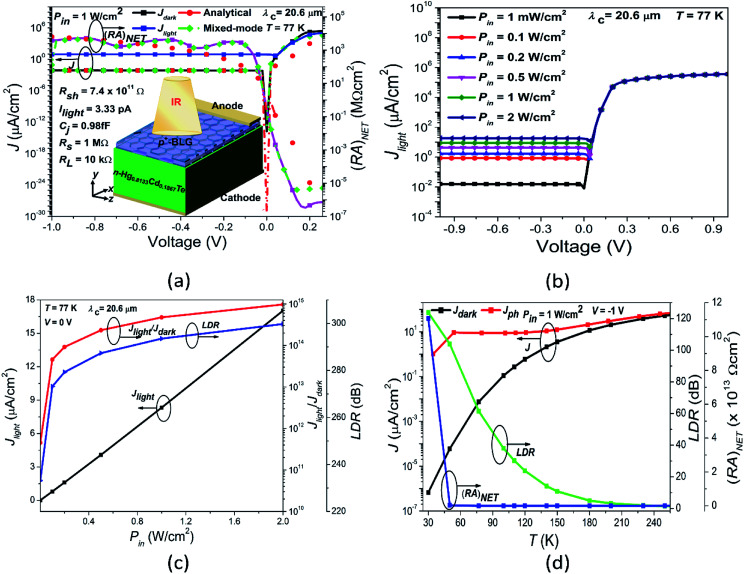
The electrical characteristics of p^+^-BLG/n-Hg_0.8133_Cd_0.1867_Te heterojunction VLWIR photodetector at a cut-off wavelength (*λ*_c_) of 20.6 µm. (a) The current density (*J*) and net resistance area product (RA)_NET_ as a function of applied voltage (*V*) at 77 K under dark and illumination conditions with *P*_in_ = 1 W cm^−2^. Here, the values of *I*_light_, *C*_j_, and *R*_sh_ are obtained at zero-bias condition and are used for Mixed-Mode simulation to evaluate device performances in the dark condition. The obtained results from Atlas and Mixed-Mode simulations are well in accordance with the results obtained from the analytical modeling. The inset shows the 3D schematic view of the photodetector. (b) The *J*_light_–*V* characteristics of the photodetector for different *P*_in_ at 77 K. (c) *J*_light_, *J*_light_/*J*_dark_ ratio, and linear dynamic range (LDR) of the photodetector as a function of *P*_in_ in self-powered mode (zero bias condition) at 77 K. The *J*_light_/*J*_dark_ ratio and LDR increases exponentially with *P*_in_, whereas, *J*_light_ increase linearly with *P*_in_. (d) The variation of current densities, LDR, and (RA)_NET_ with temperature at a bias of −1.0 V under illumination of 1 W cm^−2^. The current densities increase exponentially, whereas LDR and (RA)_NET_ decreases with the increase in temperature due to the generation of thermally induced electron–hole pairs in addition to photoexcited carriers.

Fig. 3a shows the simulated current density (*J*) as function of applied voltage varying from −1.0 to 0.3 V. The results are obtained under dark condition, and at a radiation of incident power intensity (*P*_in_) of 1 W cm^−2^ at 20.6 µm. For the Mixed-Mode simulations of the circuit shown in Fig. 1c under dark condition, the values of *I*_light_, *C*_j_, and *R*_sh_ are obtained at zero-bias condition. The proposed photodetector exhibits a low dark current density of 1.75 × 10^−14^ µA cm^−2^ and a photocurrent density of 8.33 µA cm^−2^ at zero-bias (self-powered mode) which in turn improves the signal-to-noise ratio (SNR), and hence specific detectivity. The simulated values of *J*_dark_–*V* characteristic are well in accordance with the values obtained from the analytical modeling. The obtained *J*_dark_ is smaller than that of the state-of-art photodetectors such as Hg_1−*x*_Cd_*x*_Te based IR photodetector,^24^ Gr-Silicon Schottky IR photodetector,^1^ Gr-Germanium Schottky IR photodetector,^3^ Gr-GaAs IR photodetector^39^ and armchair graphene nanoribbons IR photodetector.^83^

The *J*_light_–*V* characteristics under illumination at 20.6 µm for different *P*_in_ varying from 1 mW cm^−2^ to 2 W cm^−2^ are shown in Fig. 3b. The *J*_light_ increases with the incident power, and shifts towards the positive voltage region due to unidirectional charge transport mechanism. The two important figure-of-merits to evaluate the electrical performance of photodetectors are *J*_light_/*J*_dark_ ratio and linear dynamic range^33^ (LDR (dB) = 20 log(*J*_light_/*J*_dark_)). Fig. 3c shows the *J*_light_, *J*_light_/*J*_dark_ ratio, and LDR for various *P*_in_ values at zero-bias and 77 K. The device exhibits *J*_light_/*J*_dark_ ratio of 4.8 × 10^14^ and LDR of 293.55 dB at 2 W cm^−2^ in self-powered mode and are better than that of Gr–Si based heterojunction photodetector.^84,85^ The photocurrent density *J*_light_ and incident power *P*_in_ satisfy the relationship^86^*J*_light_ ∝ *P*_in_^*α*^, where *α* is an empirical value related to the recombination process of the photoexcited carriers. From Fig. 3c, *α* = 1.0 is obtained for reverse biases varying from −1.0 to 0.0 V. The linear response of photocurrent density as a function of incident power indicates that the recombination loss is negligible for the proposed heterojunction photodetector. Fig. 3d shows the current density, LDR, and effective or net resistance area product (RA)_NET_ with respect to temperature variation at a bias of −1.0 V and incident power of 1 W cm^−2^. The increase in temperature decreases the resistance and LDR, whereas, increases the current density. The (RA)_NET_ arises due to different current densities, *i.e.*, *J*_DIFF_, *J*_GR_, *J*_TAT_ and *J*_BTB_, and can be written as:14
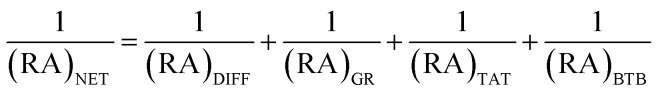


The simulated and analytical resistance area product of the proposed photodetector for different voltages varying from −1.0 to 0.3 V at 77 K is shown in Fig. 3a. The photodetector exhibits a resistance area product 
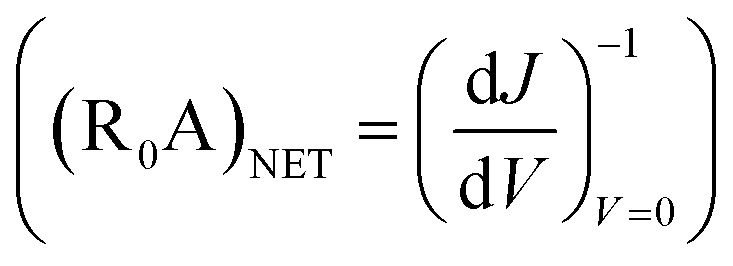
 of 0.3 MΩ cm^2^ in self-powered mode which in turn results in high specific detectivity.

### Optical characterization

The optical analysis of proposed photodetector is performed by coupling the optical and basic semiconductor equations. The optical absorption coefficient of n-Hg_1−*x*_Cd_*x*_Te material within the Kane region is calculated by Chu's empirical relation.^87,88^ For the photon energy *E*_p_ < *E*_gn_ (tail region) and *E*_p_ > *E*_gn_ (Kane region), the absorption coefficient is approximated by:15
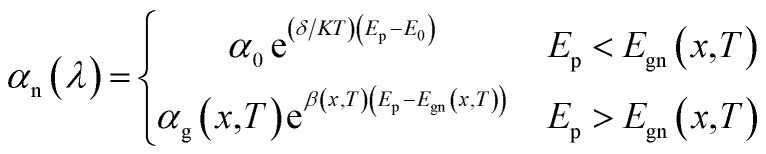
where *α*_0_ = e^(−18.5+45.68*x*)^; *E*_0_ = −0.355 + 1.77*x* the fitting parameters which vary with *x* composition and16
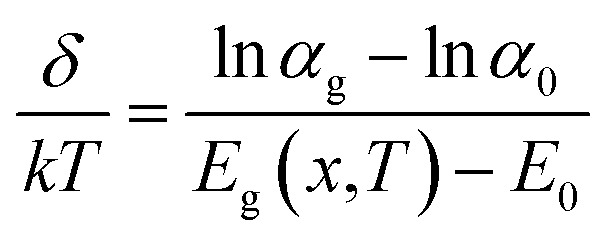
Here,

17 *α*_g_(*x*,*T*) = −65 + 1.88*T* + (8694 − 10.314*T*)*x*

18 *β*(*x*,*T*) = −1 + 0.083*T* + (21 − 0.13*T*)*x*

In order to compute the optical characteristics, complex refractive indices are described for both the BLG and MCT. The wavelength (*λ*) dependent complex refractive index for Gr is calculated by^42^*n*_Gr_(*λ*) = 3.0 + *i*1.8153*λ*, whereas, absorption coefficient is approximated by *α*_p^+^_(*λ*) = 1.8153 × 4π/*λ*. The complex refractive index of MCT as a function of *x* composition and temperature is given as:^89^19
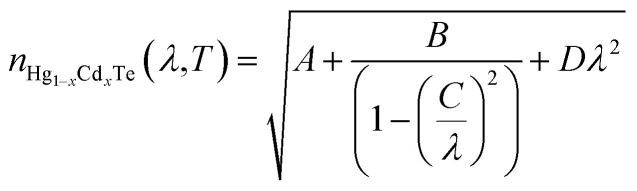
where20
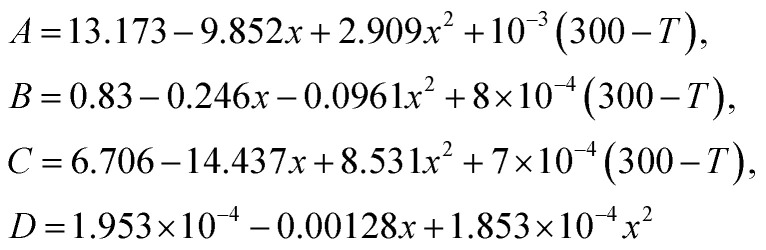


The imaginary part of the refractive index for MCT depends on the wavelength and absorption as.^24^*K* = *λα*_n_(*λ*)/4π.

The spectral response of the proposed photodetector as a function of wavelength is shown in Fig. S1.† The quantum efficiency (QE), photocurrent responsivity (*R*_i_), specific detectivity (D*), and noise equivalent power (NEP) of proposed photodetector are evaluated using computer simulations. The two types of quantum efficiencies, *i.e.* internal QE (QE_int_) and external QE (QE_ext_) are often considered for a photodetector. The QE_int_ determines the internal photocurrent responsivity (*R*^int^_i_), whereas QE_ext_ gives the external photocurrent responsivity (*R*^ext^_i_). The QE_ext_, *R*^ext^_i_, D*, and NEP of the device are further evaluated using the analytical model. The QE_ext_ measures the wavelength dependent gain of the photodetector and can be given as^32^21
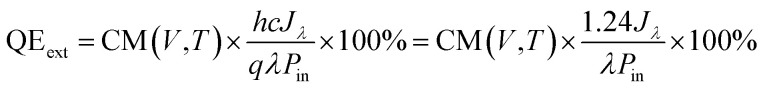
where CM represents the hot carrier multiplication factor^69–74^ which is a function of bias voltage and ambient temperature, *c* is the speed of light, *J*_*λ*_ is the photocurrent density, and *λ* represents the wavelength of the incident radiation.

The net QE_ext_ of the photodetector comprises of neutral p^+^-(QE_ext_)_p^+^_, neutral n-(QE_ext_)_n_, and the depletion (QE_ext_)_dep_ regions, and can be written as:^24,90–92^

22 QE_ext_ = CM(*V*,*T*) × ((QE_ext_)_p^+^_ + (QE_ext_)_n_ + (QE_ext_)_dep_)

where23
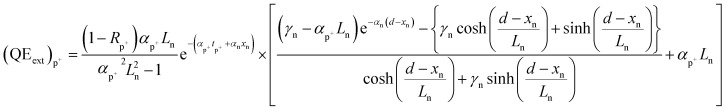
24
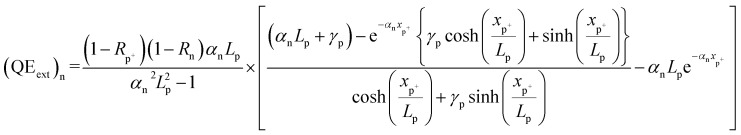
and25
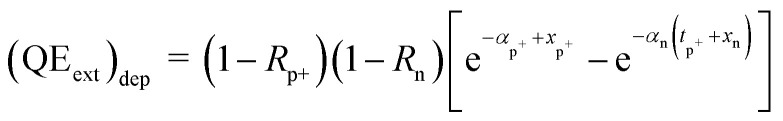
Here, *R*_p^+^_ and *R*_n_ are the Fresnel reflection coefficients at the entrance and p^+^–n interfaces, respectively.

The photocurrent responsivity is a ratio of photocurrent to the incident light power, whereas, quantum efficiency measures the sensitivity of the photodetector. On the other hand, the specific detectivity describes the smallest signal that a photodetector can detect, however, noise equivalent power is the minimum incident signal that a photodetector can resolve from the noise. The QE_int_ of the IPE process is the number of carriers emitted to HgCdTe per absorbed photon, and QE_ext_ is the number of carriers emitted to HgCdTe per incident photon. The *R*^int^_i_, *R*^ext^_i_, D*, and NEP are calculated using following relations:^3,24,32,33,93^26a
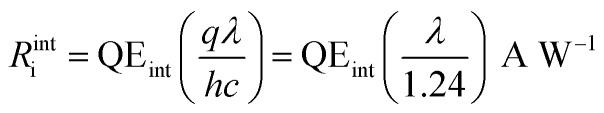
26b
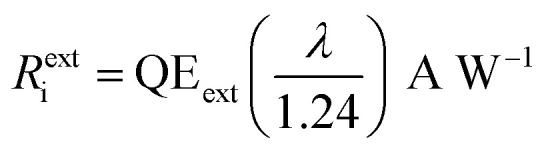
27
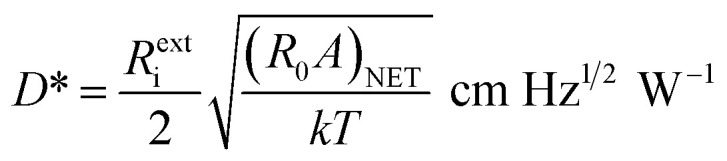
28
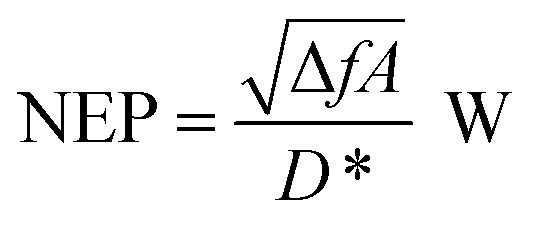
where Δ*f* and *A* are the bandwidth, and active area, respectively. Here, NEP is estimated at a unity bandwidth, *i.e.* Δ*f* = 1 Hz.

**Fig. 4:**
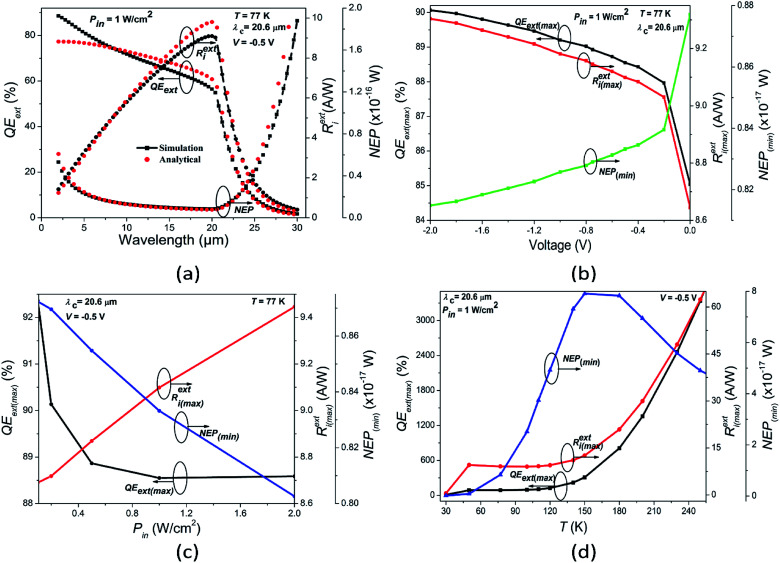
The optical characteristics of p^+^-BLG/n-Hg_0.8133_Cd_0.1867_Te photodetector at a cut-off wavelength (*λ*_c_) of 20.6 µm. (a) The external quantum efficiency (QE_ext_), external photocurrent responsivity, (*R*^ext^_i_) and noise equivalent power (NEP) as a function of wavelength with *P*_in_ = 1 W cm^−2^, *V* = −0.5 V at 77 K. The results are well in accordance with the results obtained from analytical modeling. (b) The QE_ext(max)_, *R*^ext^_i(max)_, and NEP_(min)_ as a function of bias voltage with *P*_in_ = 1 W cm^−2^ at 77 K. The QE_ext(max)_ and, *R*^ext^_i(max)_ increases with the bias voltage, whereas NEP_(min)_ decreases with the applied bias. (c) The QE_ext(max)_, *R*^ext^_i(max)_, and NEP_(min)_ of the photodetector at 77 K under different *P*_in_ values with *V* = −0.5 V. The QE_ext(max)_ and NEP_(min)_ decreases with *P*_in_, whereas *R*^ext^_i(max)_, increases with the increase in *P*_in_. (d) The variation of QE_ext(max)_, *R*^ext^_i(max)_, and NEP_(min)_ with temperature under −0.5 V bias at 1 W cm^−2^. The QE_ext(max)_ and, *R*^ext^_i(max)_ both increases with the increase in temperature, whereas NEP_(min)_ initially increases upto 150 K and improves thereafter.

Fig. 4a shows the variation of QE_ext_, *R*^ext^_i_, and NEP for the proposed photodetector as a function of wavelength at a of −0.5 V and a radiation of 1 W cm^−2^ at 20.6 µm and 77 K. The device exhibits a high QE_int_ ∼ 99.49% (Fig. S1b†), QE_ext_ ∼ 89%, *R*^int^_i_ ∼ 13.26 A W^−1^ (Fig. S1b†), *R*^ext^_i_ ∼ 9.1 A W^−1^, D* ∼ 7.6 × 10^13^ cm Hz^1/2^ W^−1^ (Fig. S2a†), and low value of NEP ∼ 8.3 × 10^−18^ W.

The influence of reverse bias voltage, incident power intensity, and temperature on the maximum external quantum efficiency (QE_ext(max)_), maximum external photocurrent responsivity, *R*^ext^_i(max)_ and minimum NEP (NEP_(min)_) under IR illumination are shown in Fig. 4b–d, respectively. The increase in reverse bias in Fig. 4b, increases the QE_ext(max)_, and *R*^ext^_i(max)_ resulting in a lower NEP. The increase in both QE_ext(max)_, and *R*^ext^_i(max)_ with the reverse bias voltage is mainly due to the increased drift velocity of the photo-induced charge carriers along with the increased probability of exciton separation and acceleration with the bias voltage, which eventually contribute to the total photocurrent in the external circuit. The increase in QE_ext_ is attributed to the carriers passing thin depletion region multiple times at a certain reverse bias.^32^Fig. 4c demonstrates the variation of incident power suggesting increase in *R*^ext^_i_ due to the increase in photocurrent which decreases both QE_ext_ and NEP. The decrease in QE_ext_ with incident power could also be attributed due to the self-induced-heating increase in the carrier scattering and hence the rate of charge carrier recombination. Furthermore, the increase in reverse bias and incident power results in improved D* as shown in Fig. S2b and c,† respectively.

The effect of temperature variation on optical parameters of the device is shown in Fig. 4d. The QE_ext(max)_ increases from 10.68 to 3337.70%, whereas the *R*^ext^_i(max)_ increases from 0.51 to 62.36 A/W for the temperatures varying from 30 to 250 K. The maximum D* 
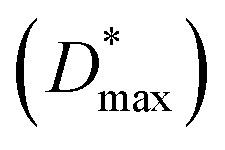
 changes from 3.8 × 10^15^ to 8.08 × 10^12^ cm Hz^1/2^ W^−1^ for temperatures 30–180 K, and from 3.04 × 10^11^ to 1.29 × 10^13^ cm Hz^1/2^ W^−1^ for the temperature range 200–250 K (see Fig. S2d†). Accordingly, NEP_(min)_ varies from 1.66 × 10^−19^ to 4.90 × 10^−17^ W for the temperature range 30–250 K as shown in Fig. 4d. The proposed photodetector demonstrates a QE_ext(max)_, *R*^ext^_i(max)_, 
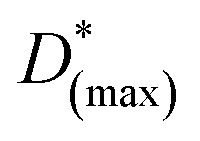
 and NEP_(min)_ of 3337.70%, 62.36 A W^−1^, 1.29 × 10^13^ cm Hz^1/2^ W^−1^ and 4.90 × 10^−17^ W, respectively, at near room temperature.

**Fig. 5:**
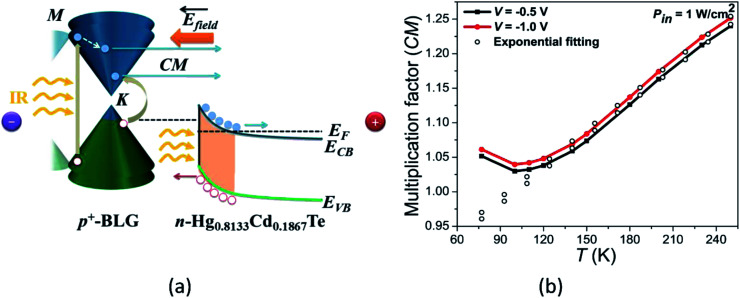
(a) The energy band diagram of p^+^-BLG/n-Hg_0.8133_Cd_0.1867_Te heterojunction based VLWIR photodetector under illumination and reverse bias condition showing carrier multiplication (CM). (b) The carrier multiplication factor as a function of temperature at −0.5 and −1.0 V under illumination of 1 W cm^−2^. The CM factor increases exponentially with the increase in temperature.

The high values of QE_ext_ more than 100% are generally associated with the well-known carrier multiplication effect in graphene.^69–74^ Such multiple hot-carrier generation can be explained with the schematic representation of energy band diagram of proposed device as shown in Fig. 5a. The incident optical energy excites an electron in the valence band to the higher conduction state of the graphene which further excites another valence band electron to conduction band as shown in Fig. 5a. Therefore, generation of multiple hot-carriers in graphene with single incident photon is due to impact ionization. Such carrier multiplication effect is observed to be increasing with doping in graphene.^74^ Moreover, under the illumination of high optical energy, light can also be absorbed in both the *M*- and *K*-points of the Brillouin zone in graphene resulting in enhanced carrier multiplication effect.^33^

The combined effect of carrier multiplication (in graphene) and presence of high electric field across the junction causes transport of multiple electrons (holes) to the positive (negative) bias electrodes which finally contributes to the net enhanced photocurrent and QE_ext_.

The CM(*V*,*T*) can be estimated from the simulated *J*–*V* characteristics under dark and illumination conditions at different temperatures as:^27^29
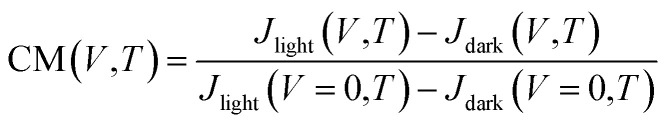


Fig. 5b shows the estimated CM factor at −0.5 and −1.0 V bias as a function of temperature. The CM factor is observed to be increasing with ambient temperature exponentially which is attributed to the temperature dependence of the semiconductor current according to eqn (29). The CM factor is observed to be varying in the range of 1.05–1.24 and 1.06–1.25 at −0.5 and −1.0 V, respectively, for the temperature range 77 to 250 K. With increasing reverse bias, the probability of carrier recombination decreases resulting in long life-time of photo-generated carriers and, hence, improves effective carrier multiplication in the device. In our case, the obtained values of hot CM factor for p^+^-doped graphene are well in accordance with the previously reported experimental values.^73,74^

**Fig. 6:**
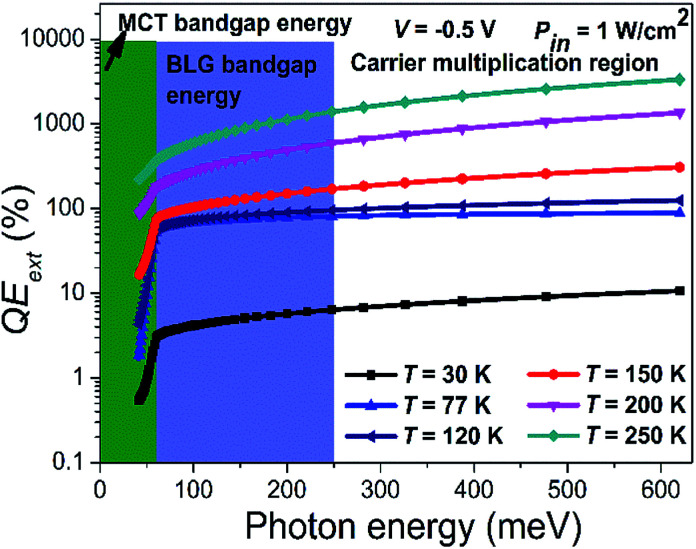
The QE_ext_ as a function of photon energy with an incident power of 1 W cm^−2^ at −0.5 V bias for different temperatures varying from 30 to 250 K. The QE_ext_ exceeds 100% due to the generation of long lifetime of photo-induced hot carriers in VLWIR region.

The variation of QE_ext_ as a function of photon energy is shown in Fig. 6. The QE_ext_ increases with the incident photon energy due to prominent hot CM under illumination with higher energy than the band-gap of BLG. On the other hand, photon energy smaller than the BLG band-gap lowers the QE_ext_ which is attributed to the decreased carrier multiplication as shown in Fig. 6. Furthermore, the external reverse bias provides a high electric field to the device which increases the probability of electron–hole pair separation and enhances drift-velocity of the photogenerated charge carriers in both graphene and MCT.

In addition, we have estimated the shot (quantum) and Johnson–Nyquist (thermal) noise,^93^ which also dominate in the photodetector. Accordingly, the shot (*i*_s_) and Johnson–Nyquist (*i*_j_) noise currents are calculated by^82^
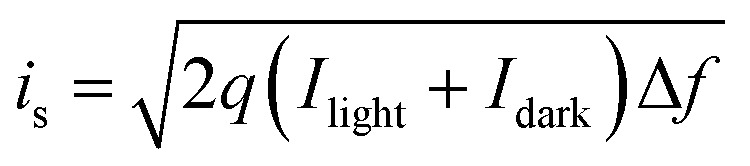
 and 
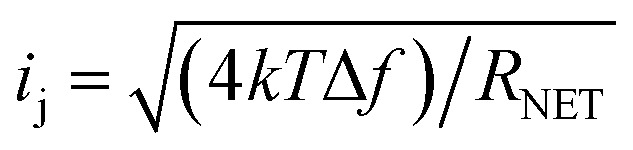
, where *I*_dark_ is the dark current *R*_NET_ = (d*I*/d*V*)^−1^, is the net resistance of photodetector in dark condition at reverse bias and Δ*f* = 1 Hz is the bandwidth. The total noise current (*i*_n_)^93^ and SNR^82^ are given by *i*_n_ = *i*_s_ + *i*_j_ and 
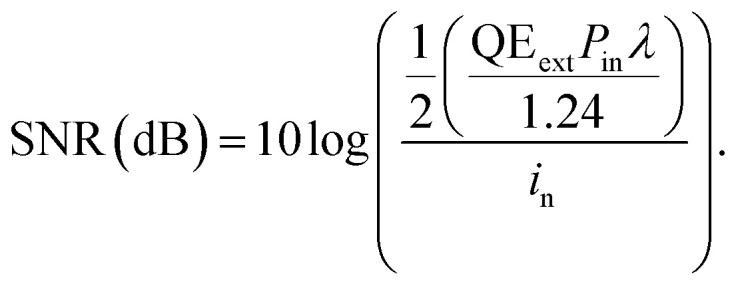


**Fig. 7:**
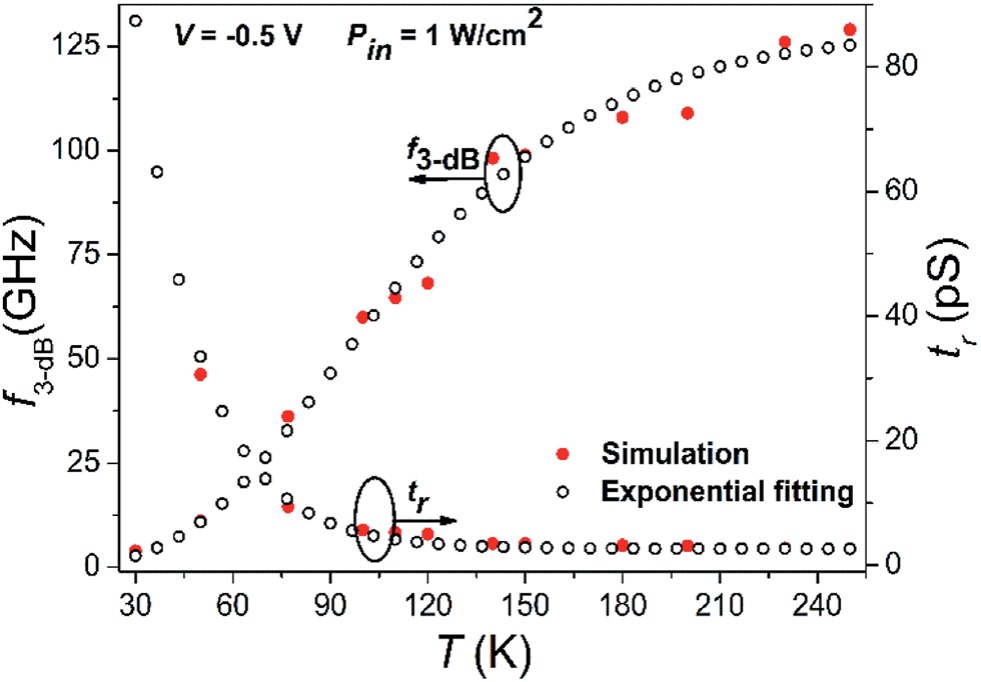
The *f*_3 dB_ and *t*_r_ as a function of temperature with *V* = −0.5 V and *P*_in_ = 1 W cm^−2^. The *f*_3 dB_ rise exponentially with temperature, whereas, *t*_r_ decay exponentially with temperature, due to increase in depletion length at higher temperatures which in turn reduces space-charge capacitance.

The estimated values of *i*_n_ and SNR are 1.06 fA and 156.18 dB, respectively, for −0.5 V bias and a radiation of 1 W cm^−2^ at 20.6 µm and 77 K. Fig. 7 shows the 3 dB cut-off frequency (*f*_3 dB_) and response time (*t*_r_) as a function of temperature at −0.5 V bias with incident power of 1 W cm^−2^. Beyond 3 dB cut-off frequency, the current/gain starts rolling-off quickly. The response time suggests the increase in output signal from 10–90% of the maximum value and is related to 3 dB cut-off as^85^30
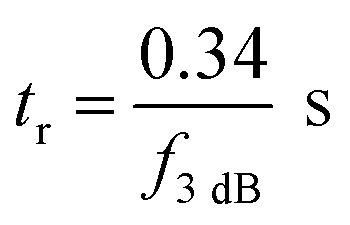


The results suggest exponential increase in 3 dB cut-off frequency from 3.89 to 129 GHz due to the existence of high saturation velocity and high carrier mobility, whereas *t*_r_ decreases from 87.4 to 2.6 ps as shown in Fig. 7. The reduction in depletion (space-charge) capacitance and increase in depletion length is responsible for obtaining larger cut-off frequency.^82^ It can be further increased with the increase in reverse bias voltage and reduction in the device active area inducing reduced capacitance. To the best of our knowledge, the obtained results for the proposed photodetector are better than that of previously reported graphene based photodetectors.^1,3,29,30,33,37,39,43,82–85,93–103^Table 2 summarizes the key parameters of our proposed device with other MCT and graphene based photodetectors reported earlier.

**Table 2:** Performance comparison of proposed device with other graphene and MCT based photodetectors reported earlier

	Device structure	QE (%)	*R*_i_ (A W^−1^)	D* (cm Hz^1/2^ W^−1^)	NEP (W Hz^−1/2^)	*J*_light_/*J*_dark_	*t*_r_	*f*_3 dB_	LDR (dB)	Ref.
Experimental Study	Graphene/Si Schottky junction	QE_int_ = 10–30	0.0028–0.0099	—	—	—	—	—	—	M. Amirmazlaghani *et al.*^1^
	Graphene/Ge Schottky junction	—	0.00518	1.38 × 10^10^	—	2 × 10^4^	23 µs	—	—	L. H. Zeng *et al.*^3^
	BLG terahertz photodetector	—	—	9 × 10^6^	—	—	—	—	—	M. Mohammadian *et al.*^29^
	Gr/SiO_2_/Si photodetector	*Q*_int_ = 6–16	0.0005	—	—	—	—	106 GHz	—	F. Xia *et al.*^30^
	Graphene/silicon Schottky junction	∼98	0.2	1.6 × 10^13^	—	1.2 × 10^6^	—	—	119	X. Wan *et al.*^33^
	Graphene nanoribbons passivated with HfO_2_	—	1.75, 1.5 and 0.18	—	—	∼7 ± 1	—	—	—	X. Yu *et al.*^37^
	BLG/GaAs Schottky junction	—	0.0012	7.3 × 10^9^	—	1.2 × 10^3^	32 µs	—	—	L. B. Luo *et al.*^39^
	BLG/GaAs Schottky junction with AlO_*x*_ interface passivation layer	—	0.005	2.88 × 10^11^	—	3 × 10^5^	320 ns	—	—	L. B. Luo *et al.*^39^
	Multilayer armchair graphene nanoribbons	—	—	2.1 × 10^11^ at 77 K and 2.2 × 10^8^ at 300 K	—	—	—	—	—	E. Ahmadi *et al.*^83^
	Graphene/silicon heterojunction	QE_ext_ = 60	0.73	4.2 × 10^12^	0.075 × 10^−12^	10^7^	320 µs	—	—	X. Li *et al.*^84^
	Graphene/Si with interfacial oxide layer	QE_ext_ = 60	0.73	5.77 × 10^13^	0.0055 × 10^−12^	10^7^	320 µs	—	90	X. Li *et al.*^84^
	Graphene/silicon Schottky junction	QE_ext_ = 60–70	0.0003	—	—	—	12 ns	2.5 MHz	—	H. Selvi *et al.*^85^
	Graphene/silicon Schottky junction	—	*R* ^int^_i_ = 0.25 *R*^ext^_i_ = 0.02	—	—	—	—	120 MHz	—	M. Casalino *et al.*^93^
	HgCdTe multilayer heterostructure	—	8.5	1.6 × 10^9^	—	—	—	0.1 GHz	—	M. Kopytko *et al.*^94^
	GaAs nanocone/MLG array Schottky junction	—	0.00373	1.83 × 10^11^	—	10^4^	72 µs	—	—	L. B. Luo *et al.*^95^
	All-carbon graphene nanoribbon-C_60_ hybrid nanostructure	—	0.4	—	—	—	4 s	—	—	X. Yu *et al.*^96^
	Graphene nanowalls/Si heterojunction	—	0.52	5.88 × 10^13^	5.96 × 10^−15^	2 × 10^7^	40 µs	8.5 kHz	—	J. Shen *et al.*^97^
	Graphene/Bi_2_Se_3_ heterostructure	—	8.18	1.7 × 10^9^	—	—	—	—	—	J. Kim *et al.*^98^
	Graphene/silicon heterojunction	QE_int_ = 65	0.435	7.69 × 10^9^	1 × 10^−12^	10^4^	1.2 ms	—	—	X. An *et al.*^99^
	MLG/InP Schottky junction	3.96	0.0461	3.62 × 10^9^	3.75 × 10^−12^	230	25.9 µs	9.2 kHz	51.7	L. B. Luo *et al.*^100^
	MLG/InP with SiO_2_ encapsulated gold nanorods	14.7	0.1398	10.5 × 10^10^	—	776	441 ns	104 kHz	—	L. B. Luo *et al.*^100^
Theoretical/simulation study	HgCdTe based heterojunction	78 at 77 K	—	4.7 × 10^12^ at 77 K	2 × 10^−12^ at 1 Hz and 77 K	—	—	—	—	P. K. Saxena *et al.*^23^
	HgCdTe based heterojunction	80 at 77 K	6.75 at 77 K	2.25 × 10^9^ at 77 K	1 × 10^−17^ at 1 Hz and 77 K	—	—	—	—	A. D. D. Dwivedi^24^
	Multiple graphene layer photodetectors	—	12 for MLG; 35–350 for 50 layers graphene	(4.1–8.2) × 10^8^ for MLG at 300 K; (1.7–3.4) × 10^9^ for 50 layers graphene at 300 K; (1.7–3.4) × 10^13^ for 50 layers graphene at 77 K	—	—	—	—	—	V. Ryzhii *et al.*^43^
	Multiple graphene layer photodetectors	4.6 for MLG; 1.8 for 100 layers graphene	227 for 100 layers graphene	10^9^ at 300 K	—	—	—	—	—	M. Ryzhii *et al.*^44^
	HgCdTe based homojunction	67 at 77 K	—	—	1 × 10^−13^ at 1 Hz and 77 K	—	—	—	—	P. K. Saxena *et al.*^82^
	MLG photodetector	QE_int_ = 2.5–10.2	8.4 × 10^−4^-3.4 × 10^−3^	—	—	—	—	—	—	Q. Gao *et al.*^101^
	Multiple graphene layer photodetectors	4.6 for MLG; 74 for 20 layers graphene 1.8 for 100 layers graphene	227 for 100 layers graphene	10^13^ at 77 K 10^9^ at 300 K	—	—	—	—	—	V. Ryzhii *et al.*^102^
	Graphene superlattice-based photodetector	20	0.866	—	—	—	—	—	—	M. Moradinasab *et al.*^103^
	p^+^-BLG/n-HgCdTe heterojunction	QE_int_ = 99.49% QE_ext_ = 89 at 77 K QE_ext_ = 3337.70 at 250 K	*R* ^int^_i_ = 13.36 *R*^ext^_i_ = 9.1 at 77 K, *R*^ext^_i_ = 62.36 at 250 K	7.6 × 10^13^ at 77 K1.29 × 10^13^ at 250 K	8.3 × 10^−18^ at 77 K and 1 Hz 4.90 × 10^−17^ W at 250 K and 1 Hz	4.8 × 10^14^	9.4 ps at 77 K 2.6 ps at 250 K	36.16 GHz at 77 K 129 GHz at 250 K	293.55	Present work

## Conclusions

In summary, we have demonstrated a high-performance p^+^-BLG and few layer graphene/n-Hg_0.8133_Cd_0.1867_Te heterojunction VLWIR photodetector. The various electrical and optical characteristic parameters are computed and analysed using computer simulations and are further validated by an analytical model based on drift-diffusion, tunneling and Chu's methods. The dark current density is reduced due to lower thermo-generation rate of BLG and MCT heterostructure, offering the high quantum efficiency, high photocurrent responsivity, high specific detectivity, low noise equivalent power, low noise current, and better signal to noise ratio. The near room temperature external quantum efficiency of 3337.70% and responsivity of 62.36 A W^−1^ at −0.5 V bias are achieved in addition to a higher 3 dB cut-off frequency (∼129 GHz) and short response time (∼2.6 ps). Such superior performances are obtained due to hot carrier multiplication mechanism in graphene. The obtained results suggest the utilisation of p^+^-BLG/n-Hg_0.8133_Cd_0.1867_Te heterostructure for next-generation high-performance, ultra-low-power, low noise, and cost effective IR photodetection.

## Conflicts of interest

There are no conflicts to declare.

## Supplementary Material

RA-008-C8RA07683A-s001
