# Supplementary material for: Bilayer graphene/HgCdTe based very long infrared photodetector with superior external quantum efficiency, responsivity, and detectivity
Source: RSC Adv. 2018 Nov 27;8(69):39579–92. doi: 10.1039/c8ra07683a (PMC9090716; doi:10.1039/c8ra07683a)
Supplement: RA-008-C8RA07683A-s001 [file RA-008-C8RA07683A-s001.pdf]

Electronic Supplementary Material (ESI) for RSC Advances.
This journal is © The Royal Society of Chemistry 2018

Electronic Supplementary Information for

Bilayer Graphene/HgCdTe Based Very Long Infrared Photodetector with Superior External Quantum Efficiency, Responsivity, and Detectivity

Shonak Bansal,[a] Kuldeep Sharma, [a] Prince Jain, [a] Neha Sardana,[b] Sanjeev Kumar,[c] Neena Gupta[a] and Arun K. Singh[a*]

[a]Department of Electronics and Communication Engineering, Punjab Engineering College (Deemed to be University), Sector–12, Chandigarh-160012, India

[b]Department of Metallurgical and Materials Engineering, Indian Institute of Technology, Ropar, India

[c]Department of Applied Sciences, Punjab Engineering College (Deemed to be University), Sector–12, Chandigarh-160012, India.

*E-mail: arun@pec.ac.in

Hole and electron effective masses

According to Kane band and Weiler model, the effective mass of hole (m_p^*) and electron (m_n^*) can be estimated by Eqns. S1 and S2, respectively[1,2]:

$$m_p^* = 0.55 * m_0 \qquad \text{(S1)}$$

$$m_n^* = \frac{m_0}{\left[-0.6 + 6.333\left(\frac{2}{E_{gn}(x,T)} + \frac{1}{E_{gn}(x,T)+1}\right)\right]} \qquad \text{(S2)}$$

where m_0 is the rest mass of electron.

Spectral response

Fig. S1a shows the spectral response of proposed photodetector which is a variation of the source photocurrent (I_S), available photocurrent (I_A) and cathode photocurrent (I_{light}). Here, I_S and I_A represent the rate of incident and absorbed photons, respectively, in the device. Typically, I_A is smaller than that of I_S due to reflection and transmission of illumination from the device. The results demonstrate rapid fall in available photocurrent and cathode current beyond cut-off wavelength, i.e., 20.6 µm for the proposed VLWIR photodetector. The internal quantum efficiency (QE_{int}) and external quantum efficiency (QE_{ext}) are estimated from the following eqns. S3 and S4[3,4]

$$QE_{int}(\%) = \left({}^{I_A}\!/\!_{I_S}\right) \times 100 \qquad \text{(S3)}$$

$$QE_{ext}(\%) = \left({}^{I_{light}}\!/\!_{I_S}\right) \times 100 \qquad \text{(S4)}$$

The variation of internal quantum efficiency (QE_{int}) and internal photocurrent responsivity $\left(R_i^{int}\right)$ as a function of wavelength at 77 K with a bias of –0.5 V is shown in Fig. S1b.

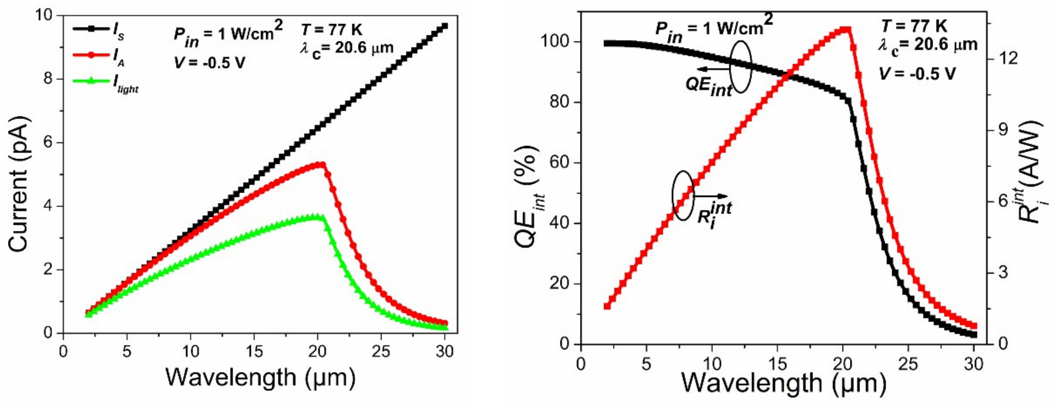

(a) **(b)**

Fig. S1. (a) The spectral response of the photodetector with P_{in} = 1 W/cm^2 and V = –0.5 V at 77 K. I_S, I_A, and I_{light} corresponds to source photocurrent, available photocurrent, and cathode photocurrent, respectively. (b) The internal quantum efficiency (QE_{int}), and internal photocurrent responsivity $\left(R_i^{\mathrm{int}}\right)$ as a function of wavelength with P_{in} = 1 W/cm^2, V = –0.5 V at 77 K. The cut-off wavelength of proposed VLWIR is 20.6 µm. The device exhibits the maximum QE_{int} and maximum R_i^{int} of 99.49% and 13.26 A/W, respectively.

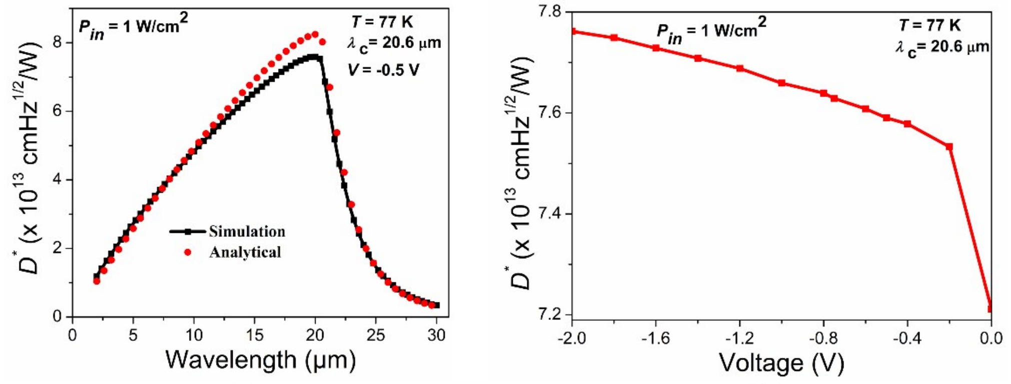

(a) **(b)**

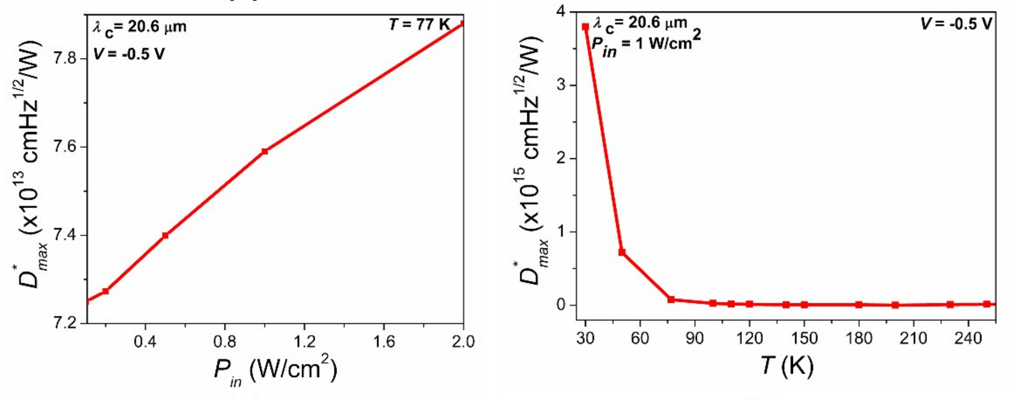

(c) **(d)**

Fig. S2. (a) The specific detectivity (D^*) as a function of wavelength with incident power of 1 W/cm^2 at 20.6 µm at V = –0.5 V and T = 77 K. The results are well in accordance with the results obtained from analytical model. The maximum D^* of photodetector is ~7.6 × 10^{13} cmHz$^{1/2}$/W at 20.6 µm wavelength. (b) The D^*_{max} value as a function of applied reverse bias voltage under 20.6 µm IR radiation with an incident power of 1 W/cm^2 at 77 K. The increase in reverse bias voltage increases the D^*_{max}. (c) The D^*_{max} at V = –0.5 V and T = 77 K of the photodetector as a function of P_{in}. The D^*_{max} value increases linearly with P_{in}. (d) The D^*_{max} value of the photodetector under different temperatures at V = –0.5 V and P_{in} = 1 W/cm^2. The D^*_{max} value decreases with temperature.

Specific detectivity (D^*)

The Fig. S2a shows the simulated and analytical results of D^* as a function of wavelength for the proposed BLG/HgCdTe photodetector at –0.5 V bias with an illumination of 20.6 µm at 1 W/cm^2 and 77 K, whereas, Figs. S2b, S2c and S2d show the maximum D^* (D^*_{max}) with respect to applied reverse bias, different incident power P_{in} and temperature, respectively.

Effect of temperature on external quantum efficiency (QE_{ext})

The QE_{ext} increases with the increase in temperature at a bias of –0.5 V as given in Fig. S3†. However, it demonstrates a different behaviour at low temperatures i.e. 30-120 K (in the inset of Fig. S3†), and at high temperatures varying from 140 to 250 K. Interestingly, QE_{ext} exceeds more than 100% for temperatures starting from 120 K due to the generation of long lifetime of photo-induced hot carriers (electron–holes) in VLWIR region. The increase in recombination of charge carriers at higher wavelength reduces the flow of photocurrent and hence decreases QE_{ext} as shown in Fig. S3†.

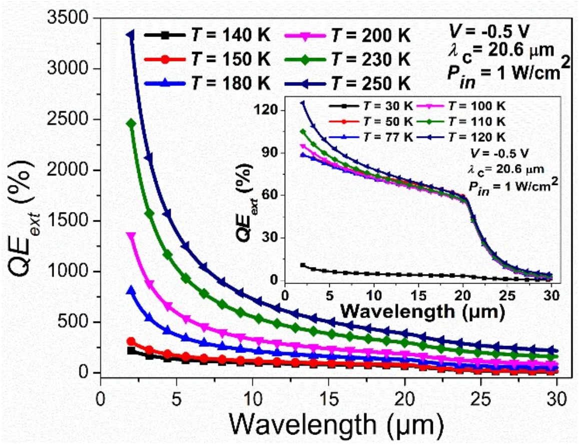

Fig. S3. The QE_{ext} as a function of wavelength with an incident power of 1 W/cm^2 at 20.6 µm for –0.5 V bias at different temperatures varying from 30 to 250 K. The QE_{ext} exceeds 100% for different temperatures from 120 to 250 K due to the generation of long lifetime of photo-induced hot carriers in VLWIR region.

References

1 A. Rogalski, *Reports on Progress in Physics*, 2005, **68**, 2267–2336.
2 M. H. Weiler, *Semiconductors and Semimetals*, 2008, **16**, 119–191.
3 T. Varma, S. Sharma, C. Periasamy and D. Boolchandani, *Journal of Nanoelectronics and Optoelectronics*, 2015, **10**, 1–5.
4 S. Sharma, A. Sumathi and C. Periasamy, *IETE Technical Review*, 2016, **34**, 83–90.
